# Structure of the Catalytic Region of DNA Ligase IV in Complex with an Artemis Fragment Sheds Light on Double-Strand Break Repair

**DOI:** 10.1016/j.str.2013.02.014

**Published:** 2013-04-02

**Authors:** Takashi Ochi, Xiaolong Gu, Tom L. Blundell

**Affiliations:** 1 Department of Biochemistry, University of Cambridge, 80 Tennis Court Road, Cambridge CB2 1GA, UK

## Abstract

Nonhomologous end joining (NHEJ) is central to the repair of double-stranded DNA breaks throughout the cell cycle and plays roles in the development of the immune system. Although three-dimensional structures of most components of NHEJ have been defined, those of the catalytic region of DNA ligase IV (LigIV), a specialized DNA ligase known to work in NHEJ, and of Artemis have remained unresolved. Here, we report the crystal structure at 2.4 Å resolution of the catalytic region of LigIV (residues 1–609) in complex with an Artemis peptide. We describe interactions of the DNA-binding domain of LigIV with the continuous epitope of Artemis, which, together, form a three-helix bundle. A kink in the first helix of LigIV introduced by a conserved VPF motif gives rise to a hydrophobic pocket, which accommodates a conserved tryptophan from Artemis. We provide structural insights into features of LigIV among human DNA ligases.

## Graphical abstract

**Graphical Abstract:**

**Figure:**
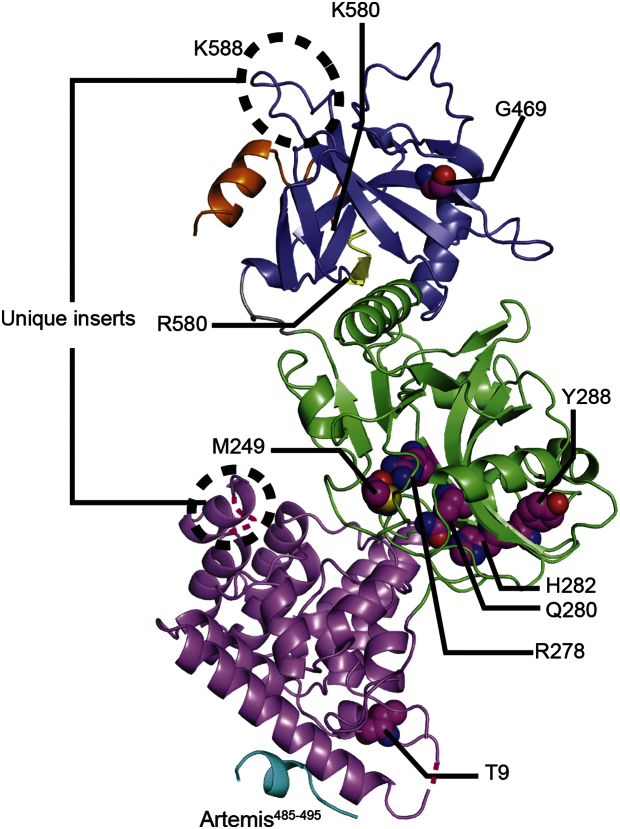

**Highlights:**

► The crystal structure of the DNA ligase IV/Artemis complex was solved at 2.4 Å ► The structure of DNA ligase IV (residues 1–609) shows inserts ► An Artemis helix forms a three-helix bundle with two helices from DNA ligase IV ► The structure provides insights into the mutations causing LIG4 syndrome

## Teaser

Nonhomologous end joining (NHEJ) is central to the repair of dsDNA breaks. Ochi et al. report the structure of the catalytic region of LigIV in complex with an Artemis fragment; this defines interactions of LigIV with Artemis and provides insights into features of LigIV that are unique among human DNA ligases.

## Introduction

DNA ligase IV (LigIV), one of three DNA ligases in higher eukaryotes, plays a central role in the repair of DNA double-strand breaks (DSBs) during nonhomologous end joining (NHEJ). In human NHEJ, two DSB ends are brought into proximity by DNA protein kinase (DNA-PK) comprising the Ku70/80 heterodimer and DNA-PK catalytic subunit (DNA-PKcs) (Smith and Jackson, 1999). The DNA ends are then processed by Artemis, PNKP, polμ, polλ, TdT, and other proteins, and end joining is achieved by the NHEJ ligase complex LigIV, XRCC4, and XLF-Cernunnos (Lieber, 2010).

Artemis, a nuclease belonging to the metallo-β-lactamase superfamily, is mutated in radiosensitive severe combined immunodeficiency patients (Moshous et al., 2001). DNA-PKcs recruits Artemis to the DNA ends through its C-terminal residues, particularly L401 and R402 (Soubeyrand et al., 2006; Niewolik et al., 2006). The complex is crucial for opening hairpin DNA in V(D)J recombination (Ma et al., 2002). Artemis is activated by the autophosphorylation of DNA-PKcs (Goodarzi et al., 2006). It has recently been found that Artemis (residues 485–495) also interacts with LigIV (Malu et al., 2012).

Human DNA ligases have a conserved catalytic region consisting of a DNA-binding domain (DBD), a nucleotidyltransferase domain (NTD), and an OB-fold domain (OBD) (Tomkinson et al., 2006). Motifs I–VI in NTD and OBD are conserved among DNA and RNA ligases and RNA-capping enzymes (Shuman and Schwer, 1995). In addition, eukaryotic DNA ligases have a further motif, Va, in OBD (Marchetti et al., 2006). A covalent AMP-lysine intermediate is formed with the catalytic lysine (K273) in motif I of LigIV (step 1) before the AMP is transferred to the 5′ phosphate of the DNA nick (step 2). The nick is then sealed by the formation of a phosphodiester bond (step 3). DNA ligase is required for all steps (Shuman and Lima, 2004).

LigIV has a tandem repeat of BRCT domains at the C terminus, which interacts with XRCC4 (Critchlow et al., 1997). The interaction stabilizes LigIV in vivo (Bryans et al., 1999) and stimulates the activities of LigIV (Grawunder et al., 1997). The knockout of either the *Lig4* or *Xrcc4* gene in mouse results in embryonic lethality (Frank et al., 1998; Gao et al., 1998). Hypomorphic mutations of *LIG4* cause a rare disease known as LIG4 syndrome (Chistiakov et al., 2009).

Although the crystal structures of the core NHEJ proteins have been defined, the structure of the catalytic region of LigIV has remained unsolved. Here, we report the crystal structure at 2.4 Å resolution of LigIV^1–609^ (residues 1–609) in complex with Artemis^485–495^ (residues 485–495). The structure shows that DNA ligases I and III share a similar fold but the catalytic domain of LigIV carries unique inserts, which might be involved in enzymatic activities of LigIV. We show that Artemis interacts with the first two α helices of LigIV, forming a three-helix bundle. A kink in the first helix of LigIV, introduced by a conserved VPF motif, gives rise to a hydrophobic pocket, which accommodates a conserved tryptophan (W489) from Artemis.

## Results and Discussion

### Purification of LigIV

**Figure 1:**
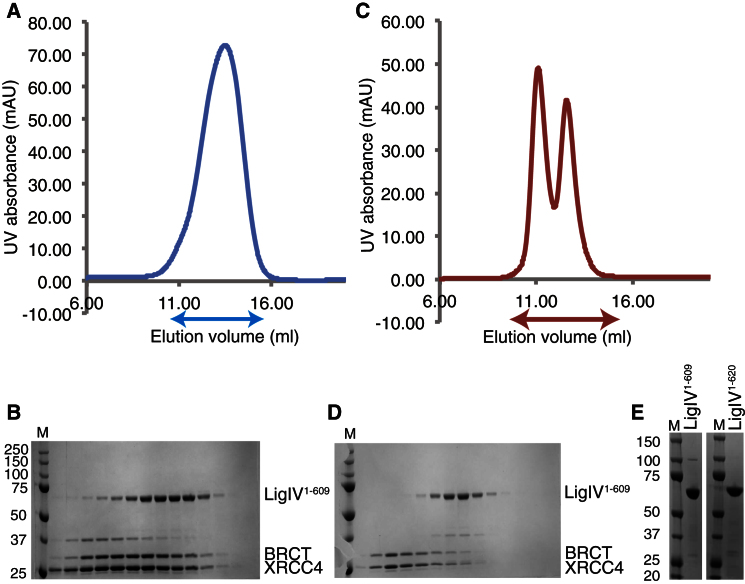
Purification of LigIV Constructs (A) Profile of the UV absorbance at 280 nm during heparin affinity chromatography. The absorbance during heparin is shown in blue. Arrows indicate the ranges of fractions used for SDS-PAGE. (B) SDS-PAGE gel of fractions eluted from a heparin column. The molecular weight markers are in column “M” and the molecular weights (kDa) of the gel are shown on the left of the gel. Protein bands are indicated on the right of the gel: LigIV^1–609^ (residues 1–609 of LigIV), BRCT (residue 645–911 of LigIV) and XRCC4 (residue 1–213 of XRCC4, all cysteines of which are mutated to alanines). (C) Profile of the UV absorbance at 280 nm during size exclusion chromatography. The absorbance during size-exclusion chromatography is shown in red. (D) SDS-PAGE gel of fractions eluted from a Superdex 200 column. The molecular weight markers are identical to those shown in (B). Protein bands are indicated on the right of the gel. (E) SDS-PAGE gels of purified LigIV^1–609^. A total of 2 μg of both LigIV^1–609^ (left) and LigIV^1–620^ (right) are shown with their molecular weights (kDa). See also Figure S1.

Possible C-terminal boundaries of the catalytic region of LigIV were initially identified as residues 620, 647, and 653 from a structure-based sequence alignment (Figure S1A available online) and these were used to guide constructs for expression of soluble protein in *E. coli* with glutathione S-transferase, maltose-binding protein, and hexahistidine tags. However, this approach proved unsuccessful (data not shown), and we explored an alternative experimental strategy. LigIV/XRCC4^ΔCTD;CtoA^ complex, in which the C-terminal region of XRCC4 (residues 214–334) is omitted and all cysteines are mutated to alanines (Ochi et al., 2012), was purified and digested with four different proteases: trypsin, chymotrypsin, papain, and subtilisin. We found that the catalytic region of LigIV remained intact after being digested with subtilisin, but less so with the other enzymes (Figure S1B), and was stable and soluble after being purified from the digested complex using heparin and gel filtration columns (Figures 1A–1D). Using mass spectroscopic analysis of an SDS-PAGE gel of the catalytic region of LigIV, the longest polypeptide observed comprised residues 1–609 (data not shown). However, later we found that constructs of LigIV comprising residues 1–609 (LigIV^1–609^) and residues 1–620 (LigIV^1–620^) were soluble in *E. coli* cells when expressed with the N-terminal Sumo tag (Figure 1E). Interestingly, the results of DNA ligation assays suggest that the residues 610–620 are important for the enzymatic activity of LigIV (Figure S1C). A recent report also suggests that the C-terminal helix of an archaeal DNA ligase is important for its activity (Tanabe et al., 2012).

### Structure of LigIV^1–609^/Artemis^485–495^ Complex

The crystallographic structure of Artemis^485–495^ in complex with mercury-labeled LigIV^1–609^, in which nine mercury atoms are coordinated by cysteines and methionines (Figure S2A), was determined at 2.4 Å resolution (Table 1) using the multiwavelength anomalous dispersion (MAD) method. The model of the protein obtained from these mercury-labeled crystals was refined against the X-ray data from the unlabeled protein crystals at 2.55 Å resolution (see Table 1 for refinement statistics). We also solved the structure of LigIV^1–609^ without Artemis^485–495^ at 2.84 Å resolution. The structures proved to be very similar except for the conformation of the loop between β11 and β12, which is distorted by the binding of thiomersal in the mercury derivative (data not shown).

**Figure 2:**
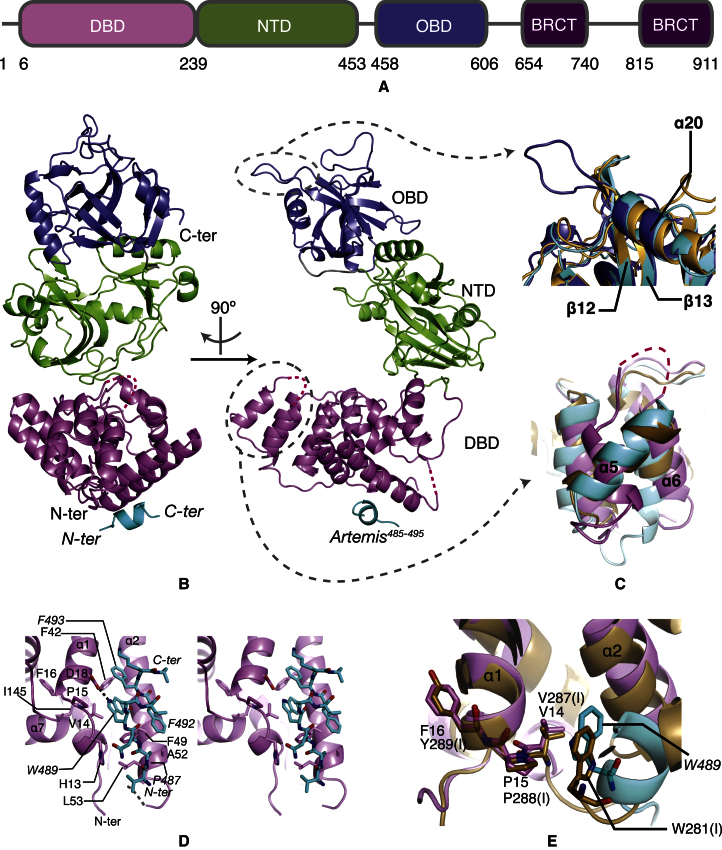
Structure of the LigIV^1–609^/Artemis^485–495^ Complex (A) Schematic representation of the domains of LigIV. The same color scheme for the domains is used in all figures throughout this paper. (B) Overall structure of LigIV^1–609^. Missing parts are represented by dotted lines. (C) Unique inserts of LigIV^1–609^ and comparisons with LigI and LigIII. Top: an insert in OBD (blue) is shown. LigI and LigIII are shown in gold and cyan, respectively. Bottom: the difference in orientation of the α5 is shown. The loop between α5 and α6, shown as a pink dotted line, is disordered in the crystal structure of LigIV^1–609^. (D) Stereo image of LigIV^1–609^ with the Artemis^485–495^. The first 53 residues of LigIV^1–609^ (pink) are shown with Artemis^485–495^ (cyan). A disordered loop between α2 and α3 is represented by a gray dotted line. A hydrogen bond between D18 and *W489* is shown by a black dotted line. Residues of LigIV^1–609^ are labeled in regular characters, whereas those of Artemis^485–495^ are in italic. (E) Conserved interaction motif VP(F/Y) in LigIV^1–609^ and LigI. The color scheme of LigIV^1–609^/Artemis^485–495^ is the same as in (D). LigI is shown in gold. Residues with “(I)” are from LigI. See also Figure S2.

The overall folds of the three catalytic domains are similar to those of human DNA ligases I and III and archaeal DNA ligases (Tomkinson et al., 2006) (Figures 2A and 2B). The first five residues, two loops (residues 58–59 and 115–123), and the last three residues of LigIV^1–609^ are disordered in the crystals. Unique structural features of LigIV^1–609^ are inserts between α5 and α6 of DBD (Insert1; residues 111–121), α15 and α16 of NTD (Ochi et al., 2012), β12 and β13 of OBD (Insert2; residues 490–494) and a different orientation of α5 of DBD (Figure 2C). In the crystals, the structure of LigIV^1–609^ is captured in an open conformation and probably stabilized by burying a surface of area 1,104 Å^2^ between OBD and NTD. This differs from other open conformations of archaeal DNA ligases available in the Protein Data Bank (PDB) (Pascal et al., 2006; Petrova et al., 2012), the buried areas of which are 337 and 80 Å^2^, respectively. Given that the archaeal structures have much smaller buried areas, they are likely to be more flexible than human LigIV and the observed conformers may be stabilized by crystallographic contacts.

In the derivative crystals of the LigIV^1–609^/Artemis^485–495^ complex, the poor density for adenosine is consistent with partial adenylation as observed by DNA ligation assay (Figure S1C). Although density for the αPO_4_ is evident, it is likely that AMP has low occupancy (Figure S2B). In nonderivative crystals, the electron density of adenosine is clearer in the catalytic pocket of LigIV^1–609^ molecules (Figure S2C). In addition, extra density near the adenosine is not explained by fitting AMP molecules, indicating that ATP may bind to LigIV^1–609^ molecules that are not adenylated. The density of β and γ phosphates is absent in the derivative crystals, probably because ATP diffuses from the binding site while the crystals are soaking in thiomersal solution, as observed by others (Håkansson et al., 1997; Pascal et al., 2006).

### Interaction between LigIV^1–609^ and Artemis^485–495^

Artemis^485–495^ interacts with α1 and α2 of LigIV to form a three-helix bundle, burying 436 Å^2^ of the surfaces of LigIV^1–609^ and Artemis^485–495^ (Figure 2B). α1 has a kink produced by the proline P15 (Figure 2D), which is conserved in higher eukaryotic organisms (Figure S2E). *W489* of Artemis (italic font for the residues of Artemis), which is an important conserved (Figure S2D) residue for LigIV interaction (Malu et al., 2012), makes a hydrogen bond from its Nε to the side chain of D18 of LigIV. It also makes van der Waals contacts with the conserved V14 in a hydrophobic pocket of LigIV, the conformation of which is constrained by I145 in α7 of LigIV (Figure 2D). The interaction of the side chain of *W489* of Artemis in the hydrophobic pocket appears to be stabilized by further interactions of *F492* of Artemis with F49 of LigIV and *F493* of Artemis with F42 of LigIV (Figure 2D), consistent with the earlier report that *W489*, *F492,* and *F493* mutations to alanines disrupt the interaction between Artemis and LigIV (Malu et al., 2012). Additionally, *P487* is localized in a hydrophobic pocket formed by F49, A52, and L53 (Figure 2D). After we submitted our manuscript, De Ioannes et al. reported the structure of DBD of LigIV in complex with Artemis^485–495^ (De Ioannes et al., 2012). Comparison of the two structures indicates very similar interactions between LigIV and Artemis (the root-mean-square deviation [rmsd] of Cα atoms of the structures is 0.975 Å calculated using LSQKAB in CCP4; Kabsch, 1976). However, the rmsd of their and our structures without Artemis is 6.88 Å, which is mainly a consequence of a difference in the conformations of α2 and the region after α10 (Figure S2F). However, the conformations of α2 with and without Artemis^485–495^ in our structures are very similar (Figure S2F). The difference between the two studies is probably due to the presence in our structure of NTD, which stabilizes the conformation of the region after α10 via contacts with α11, the following loop and that connecting α12 and NTD. α2 interacts with this region and its conformation is likely affected by it.

There are about 100 residues between the DNA-PKcs and LigIV binding regions of Artemis, so both LigIV and DNA-PKcs may bind Artemis simultaneously. Interestingly, the polypeptide sequence of the DNA-PKcs-interaction region of Artemis is not as well conserved over evolutionary time as that of the LigIV-interaction region (Figure S2D). This indicates that LigIV probably does recruit Artemis without DNA-PKcs in some organisms during DNA repair. The interaction would increase the local concentration of the interaction partners and allow repair of DSBs to occur more rapidly and efficiently.

Intriguingly, a similar interaction between LigIV and Artemis can be found within LigI, where residues Y273(I) to G284(I) [“(I)” for the residues of LigI] occupy similar positions to residues of the Artemis peptide (Pascal et al., 2004). α1 of LigI also has a kink introduced by the presence of a proline [P288(I)]. Furthermore V287 has van der Waals contacts with W281(I) (Figure 2E) that are similar to those mediating the interaction between LigIV and Artemis. The conserved VP(F/Y) is thus a tryptophan interaction motif in LigI and LigIV.

### Insights into Adenylation and DNA Ligation

**Figure 3:**
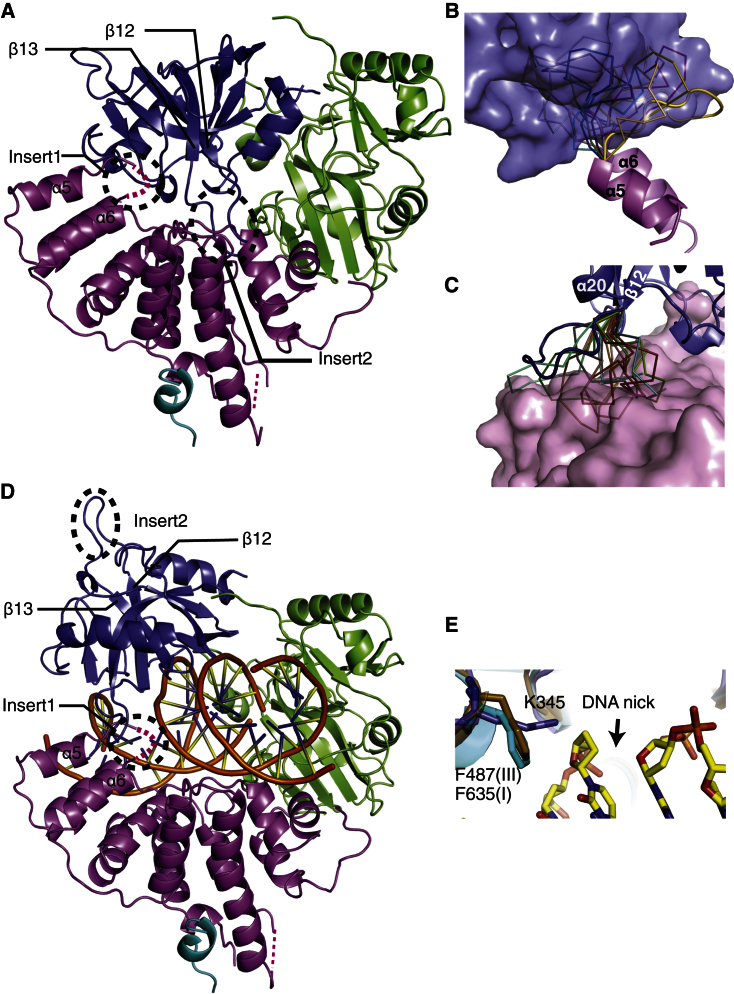
Structural Models of Closed and DNA-Bound Conformations of LigIV^1–609^ (A) Model of the closed conformation of LigIV^1–609^. Insert1 (residues 111–121) and Insert2 (residues 490–494) are circled with black dotted lines. (B) Ten RapperTK models of Insert1. The modeled loop that has the least contact with OBD (surface presentation) is shown in cartoon representation; the others are displayed in ribbon representation. (C) Ten RapperTK models of Insert2. The modeled loop that has the least contact with DBD (surface presentation) is shown in cartoon representation; others are displayed in ribbon representations. (D) Model of the DNA-bound conformation of LigIV^1–609^. DNA is from the structure of human LigIII (PDB code 3L2P). (E) DNA-bound model of LigIV^1–609^ (blue) compared with the structures of LigI (gold) and LigIII (cyan). Residues shown are key residues interacting with the base sugar of the 3′ end of the DNA nick.

OBD undergoes a large conformational change so that motif VI in OBD can be proximal to the catalytic pocket and is able to adenylate the catalytic lysine in DNA ligases (Pascal et al., 2004). In order to obtain insights into the mechanism of LigIV, the closed conformation of LigIV was modeled by superimposing each domain onto the structure of *P. furiosus* DNA ligase (Nishida et al., 2006). In the initial model, Insert1 between α5 and α6 of DBD clashed with OBD (Figure 3A). Also, the insert between β12 and β13 (Insert2) clashed with α10 and α11 of DBD (Figure 3A). In order to investigate conformations that the loops might take in the closed conformation, ten ab initio models of Insert1 and Insert2 were built using RapperTK (Gore et al., 2007). This demonstrated that Insert1 can take various conformations but they must be oriented away from the catalytic pocket in order to avoid contacts with OBD (Figure 3B). In a similar way, Insert2 must be positioned away from the catalytic pocket (Figure 3C). These results indicate that very few conformations of Insert1 and Insert2 are compatible with the closed conformation and that it may be more difficult to achieve in LigIV.

A model of the LigIV^1–609^/DNA complex was also built from the structures of LigI and LigIII (Pascal et al., 2004; Cotner-Gohara et al., 2010) (Figure 3D). Although key catalytic residues involved in ligation are conserved among human DNA ligases (Pascal et al., 2004; Cotner-Gohara et al., 2010), LigIV does not have the phenylalanine that exhibits π stacking with the ribose of the 3′ side of the DNA nick (Figure 3E). Instead, LigIV has K345, a residue that is conserved from human to fly (Figure S2E). Therefore, K345 allows more flexibility in the 3′-end detection. Another unique feature of the DNA-bound model of LigIV is that Insert1 is located close to DNA, suggesting that it may be involved in DNA binding. These unique features might assist LigIV ligate atypical substrates (e.g., a DNA nick with a gap, mismatched DNA, and poly-T ssDNA) (Gu et al., 2007a, 2007b).

### LIG4 Syndrome Mutations

**Figure 4:**
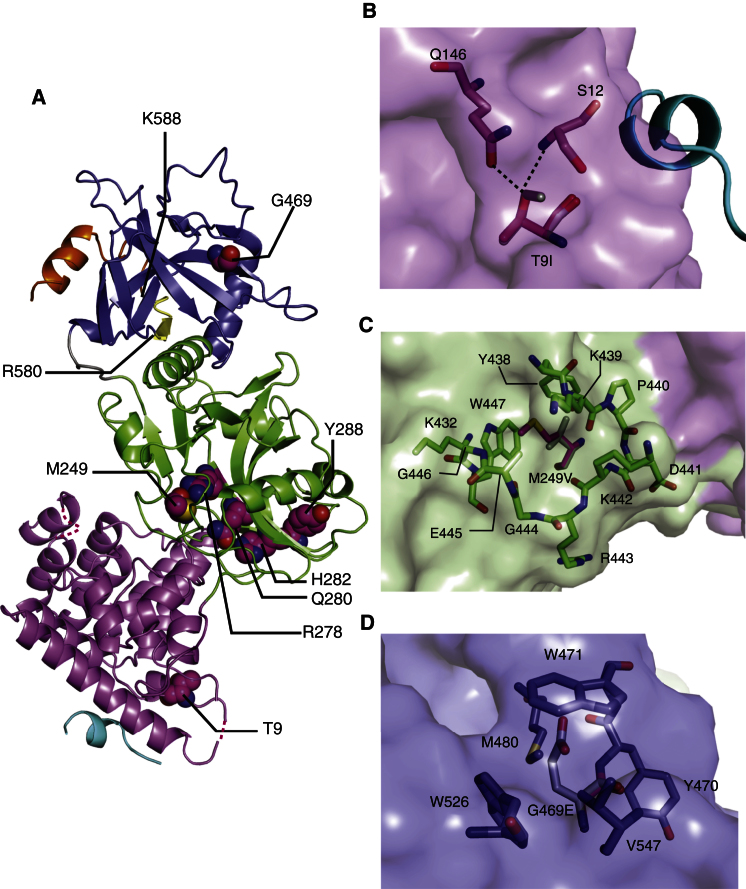
LIG4 Syndrome Mutations in the Structure of LigIV^1–609^ (A) Map of residues related to LIG4 syndrome. Residues, mutations of which cause LIG4 syndrome, are shown in a stick representation and magenta. The residues after R580 and R588 are colored in yellow and orange. (B) Mutation T9I. I9 (gray) is superimposed onto T9 (magenta). S12 and Q146 are residues having hydrogen bonds with T9. The Artemis peptide is shown in a cartoon representation. (C) Mutation M249V. V249 (gray) is superimposed onto M249 (magenta). (D) Mutation G469E. E469 (gray) is superimposed on G469.

We have previously shown that LIG4 syndrome mutations R278H, Q280R, H282L, and Y288C have structurally important consequences (Ochi et al., 2012). The complete model of NTD in this study is consistent with this conclusion. Because residues 1–5 are disordered in the crystals, the structure provided no insights into how A3V is related to LIG4 syndrome. However, the structure of LigIV^1–609^ defines the locations of T9, M249, and G469, the heterozygous mutations of which cause LIG4 syndrome (O’Driscoll et al., 2001; Toita et al., 2007) (Figure 4A). Potential effects of the mutations were assessed using Modeler to build structures of the mutants (Sali and Blundell, 1993). T9 makes polar contacts with S12 and Q146 (Figure 4B). The loss of the interactions in the T9I mutation may increase the flexibility of the residues preceding I9. M249 stabilizes the conformation of W447, which is a part of the catalytic pocket and interacts with K432, restraining its position (Figure 4C). K432, a residue in motif IV, directly interacts with the N1 atom of the adenine (Shuman, 2009). Therefore, the M249V mutation most likely leads to instability of the catalytic pocket due to the increased flexibility of K432 and W447. Because G469 is buried between tryptophan side chains, the G469E mutation leads to stereochemical clashes (Figure 4D) and likely gives rise to conformational changes in the structure of OBD, which may affect adenylation and ligation of LigIV.

## Experimental Procedures

### Constructs and Protein Purification

The DNA sequences encoding human LigIV^1–609^ and LigIV^1–620^ were amplified from the coexpression vector of LigIV/XRCC4^ΔCTD;CtoA^ (Ochi et al., 2012) and cloned between KpnI and HindIII sites of the pOPINS vector (a generous gift from Dr. Ravi Nookala and Angela Pacitto), which has an N-terminal hexahistidine followed by a Sumo tag. The purification of the proteins was performed using nickel affinity, heparin, hydrophobic interaction, and size exclusion columns. See Supplemental Experimental Procedures for further details and for purification of LigIV^1–609^ after proteolysis of LigIV/XRCC4^ΔCTD;CtoA^. Artemis^485–495^ was synthesized by Mr. Mike Waldon at the PNAC facility, Department of Biochemistry, University of Cambridge. The construct and purification of the LigIV/XRCC4 complex was described elsewhere (Ochi et al., 2012).

### Crystallization and Structural Determination

LigIV^1–609^ with and without Artemis^485–495^ was crystallized using vapor diffusion method in 100 mM MES (pH 5.6–5.7), 2.0 M (NH_4_)_2_SO_4_, 10 mM YCl_3_. Crystals appeared in 1 day and reached maximum size in 3 days. For the native data, the crystals were transferred to a cryosolution (27% [v/v] glycerol, 73% [v/v] reservoir) before being frozen in liquid nitrogen. For the MAD data, the crystals were soaked in 1 mM thiomersal for 120 min and then back soaked in the crystallization solution for 150 min. The derivative crystals were then frozen in liquid nitrogen after being protected with the cryo solution. See Supplemental Experimental Procedures for more details regarding crystallization.

X-ray data sets of the LigIV^1–609^/Artemis^485–495^ complex and LigIV^1–609^ were collected at Diamond (Oxford, UK) synchrotron radiation source and European Synchrotron Radiation Facility (Grenoble, France), respectively. The data of the complex were processed using *iMOSFLM* (Battye et al., 2011) and then scaled using Pointless and Scala (Evans, 2006) or Aimless (Evans, 2011). The data of LigIV^1–609^ were processed and scaled using XDS (Kabsch, 2010). The phenix.autosol module of PHENIX suite (Adams et al., 2010) was used to calculate experimental phases from the thiomersal data using MAD methods. The initial model was built using the phenix.autobuilt module, which created 80% of the polypeptide chains. The model was manually modified using Coot (Emsley et al., 2010) and refined using the phenix.refine module. These steps were repeated several times. The refined-protein model determined by MAD with the thiomersal data was used as the probe for molecular replacement with the wild-type and LigIV^1–609^ without Artemis^485–495^ data using the phenix.phaser module and refined as described above for the thiomersal data. The figures illustrating structures were created using PyMOL (PyMOL Molecular Graphics System version 1.2r1, Schrödinger). Buried surface areas were calculated using protein interfaces, surfaces, and assemblies service (PISA) at European Bioinformatics Institute (http://www.ebi.ac.uk/pdbe/prot_int/pistart.html).

### Modeling

Models of Insert1 and Insert2 were built using RapperTK (Gore et al., 2007). The structure of LigIV^1–609^ was used as the input and then ten loops for each insert were generated without using the restraint of electron density. Modeler (Sali and Blundell, 1993) was used to create a model of the protein with the mutations causing LIG4 syndrome. The best model was that with the lowest object function selected from 30 generated models.

### DNA Ligation Assays

See Supplemental Experimental Procedures for details regarding the DNA ligation assays.

## Figures and Tables

**Table 1:** Statistics of X-Ray Diffraction Data and Refinement Statistics of the Structures of LigIV^1–609^ and the LigIV^1–609^/Artemis^485–495^ Complex

Crystal	LigIV^1–609^ without Artemis^485–495^	LigIV^1–609^ with Artemis^485–495^			
		Native	Thiomersal		
			Peak	Inflection	Remote
Beamline	ESRF ID29	Diamond I04	Diamond I04	Diamond I04	Diamond I04
Wavelength (Å)	0.9840	0.9795	1.0036	1.0093	0.9915
Resolution (Å)	200-2.84	68.26-2.55	59.77-2.40	59.82-2.40	59.81-2.40
Space group	*P*2_1_	*P*22_1_2_1_	*P*22_1_2_1_	*P*22_1_2_1_	*P*22_1_2_1_
Cell (Å)					
a	68.29	68.26	68.57	68.63	68.60
b	104.36	105.48	105.14	105.21	105.16
c	120.04	121.08	121.98	122.04	122.14
β (°)	94.08	90	90	90	90
No. of unique reflections	39,757	27,784	34,874	34,931	34,946
Completeness (%) (highest shell)	99.4 (99.9)	96.0 (97.5)	99.3 (100)	99.3 (100)	99.3 (99.9)
Redundancy	3.4	2.8	3.5	3.5	3.5
*R*_merge_^a^ (%)	9.7 (56.8)	8.5 (56.2)	10.2 (52.8)	8.7 (53.6)	9.2 (54.8)
(highest shell)					
I/σ	10.1 (2.1)	8.8 (2.0)	8.1 (2.2)	8.5 (1.9)	8.5 (1.9)
(the highest shell)					
Resolution (Å)	47.84-2.84	59.48-2.55	59.84-2.40		
Phasing method	Molecular replacement	Molecular replacement	MAD		
FOM	N/A	N/A	0.28		
Overall score	N/A	N/A	48.25		
*R*_cryst_^b^	19.35 (25.08)	18.00 (26.64)	17.63 (24.77)		
(highest shell)					
*R*_free_^c^	24.55 (33.56)	23.36 (33.96)	22.53 (31.77)		
(highest shell)					
Rmsd					
Bond (Å)	0.009	0.008	0.008		
Angle (°)	1.252	1.153	1.129		

N/A, not applicable.

a *R*_merge_ = Σ_h_|I_h_ − 〈 I 〉 |/Σ_h_I_h_, where I_h_ is the intensity of reflection h and 〈 I 〉 is the mean intensity of all symmetry-related reflections.

b *R*_cryst_ = Σ||*F*_obs_|−|*F*_calc_||/Σ|*F*_obs_|, where *F*_obs_ and *F*_calc_ are observed and calculated structure factor amplitudes.

c As for *R*_cryst_ but using a random subset of the data (about 5%) excluded from the refinement.

## Accession Numbers

The coordinates and structure factors of the LigIV^1–609^/Artemis^485–495^ complex and LigIV^1–609^ have been deposited in the PDB with the accession numbers 3W1B, 3W1G, and 3W5O, respectively.

## Supplemental Information

Document S1. Figures S1 and S2 and Supplemental Experimental Procedures
