# Supplementary material for: Structure of the Catalytic Region of DNA Ligase IV in Complex with an Artemis Fragment Sheds Light on Double-Strand Break Repair
Source: Structure. 2013 Apr 2;21(4):672–9. doi: 10.1016/j.str.2013.02.014 (PMC3664939; doi:10.1016/j.str.2013.02.014)
Supplement: Document S1. Figures S1 and S2 and Supplemental Experimental Procedures [file mmc1.pdf]

Structure, Volume *21*

Supplemental Information

Structure of the Catalytic Region of DNA Ligase IV in Complex with an Artemis Fragment Sheds Light on Double-Strand Break Repair

Takashi Ochi, Xiaolong Gu, and Tom L. Blundell

Inventory of Supplemental Information

- **Supplemental Figures**

Suppl. Figure S1. Shows additional data for Figure 1: a sequence alignment of DNA ligases, an SDS-PAGE gel of the proteolysis of LigIV/XRCC4$^{\Delta CTD;CtoA}$ and an agarose gel of DNA ligation assays of LigIV/XRCC4, LigIV$^{1\text{-}620}$ and LigIV$^{1\text{-}609}$.

Suppl. Figure S2. Shows additional data for Figure 2: anomalous difference map & structure of the LigIV$^{1\text{-}609}$/Artemis$^{485\text{-}495}$ complex, experimentally-phased map of the LigIV$^{1\text{-}609}$/Artemis$^{485\text{-}495}$ complex & the structure of αPO_4 covalently attached to K273, electron density map & the structure of ATP, sequence alignments of Artemis & DNA ligases and the comparison of the structures of the LigIV$^{1\text{-}609}$/Artemis$^{485\text{-}495}$ complex, LigIV$^{1\text{-}609}$ & DBD (PDB code: 4HTO).

- **Supplemental Experimental Procedures**

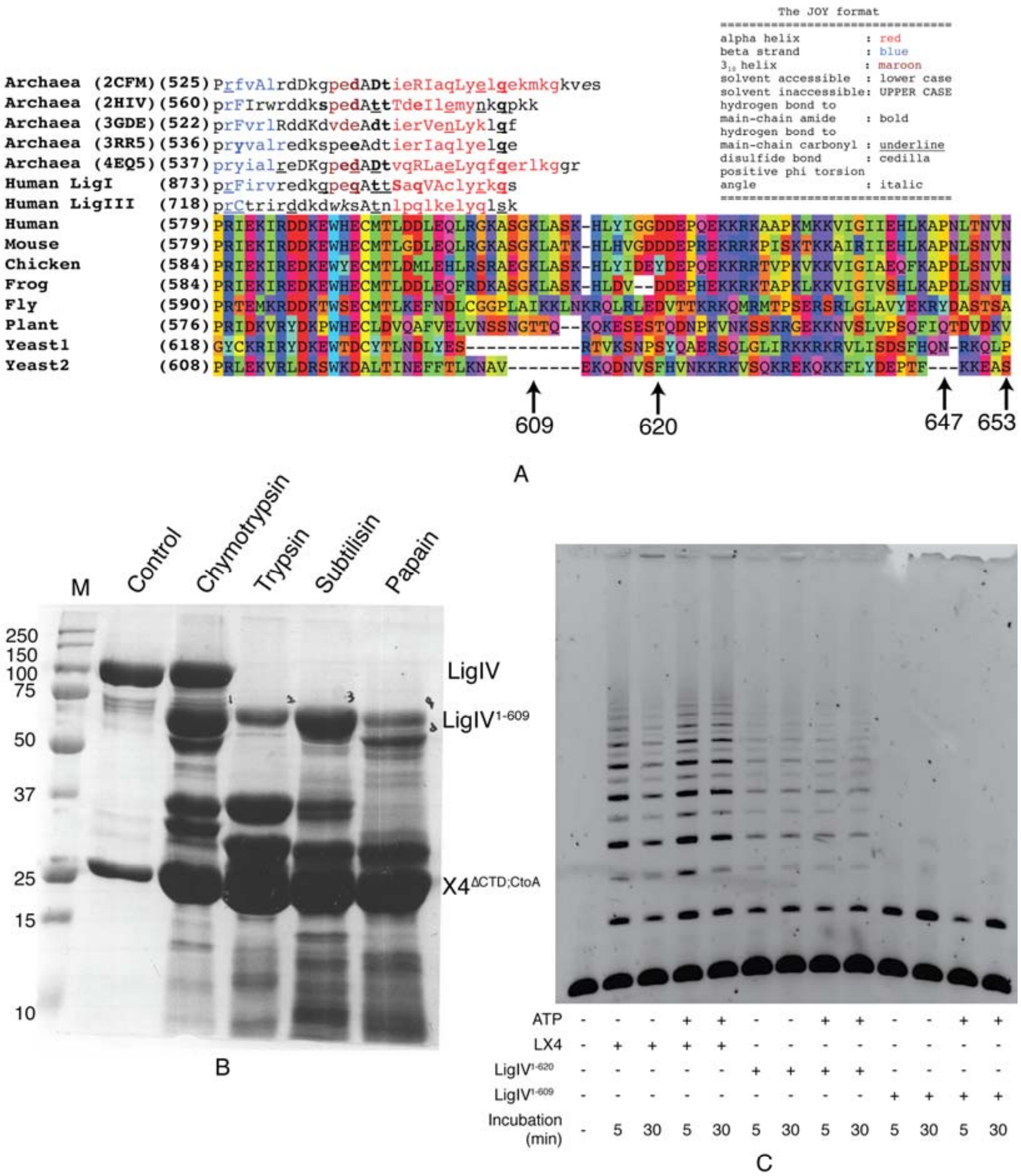

Figure S1, related to Figure 1. Purification of LigIV$^{1\text{-}609}$. (A) Structure-based sequence alignment of LigIV and DNA ligases. Sequences from structures of DNA ligases are shown in JOY format (Mizuguchi et al., 1999; please see the upper right corner for the details). The sequences with names of organism are LigIV, and archaeal DNA ligases are labeled with PDB code and residues numbers in brackets. Potential domain boundaries of human LigIV are indicated by arrows and numbers. (B)

Proteolysis of LigIV/XRCC4$^{\Delta CTD;CtoA}$. Molecular weight (kDa) markers "M" and undigested LigIV/XRCC4$^{\Delta CTD;CtoA}$ "Control" and digested samples are shown. Proteases used for the proteolysis are shown on the top of the gel. The names of identified proteins are indicated on the right of the gel. (C) DNA ligation assays of LigIV. The gel shows ligation products produced by LigIV/XRCC4 (LX4), LigIV (residue 1-620)(LigIV$^{1\text{-}620}$) and LigIV (residue 1-609)(LigIV$^{1\text{-}609}$).

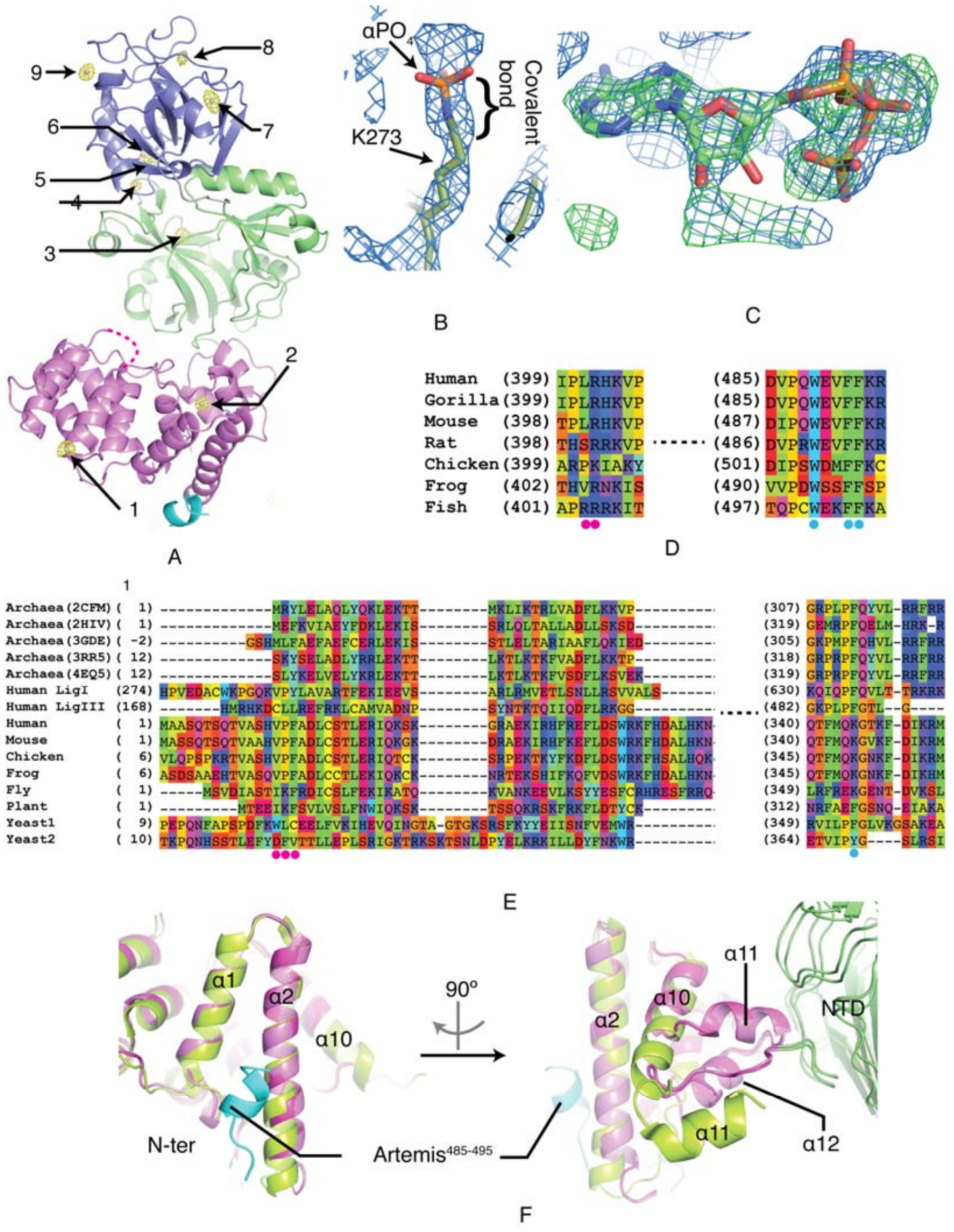

Figure S2, related to Figure 2. Structure and sequence alignment of the LigIV$^{1-609}$/Artemis$^{485-495}$ complex. (A) Anomalous difference map of the LigIV$^{1-609}$/Artemis$^{485-495}$ complex. Nine mercury sites are shown in yellow (σ=5.0). (B) Density that probably corresponds to αPO_4, covalently attached to K273. The electron density map is from the thiomersal-derivative crystals, phased using the MAD method (σ=1.5). (C) Electron density of ATP. ATP is shown with $2f_o$-f_c and f_o-f_c maps (σ=1.0

and 3.0 respectively). The difference map was calculated from the native data using the refined molecular-replacement model before adding any small molecules. (D) Sequence alignment of the DNA-PKcs and LigIV interaction surfaces of Artemis. The residue numbers are shown in brackets. The sequence alignment was carried out using Muscle (Edgar, 2004) and represented in JOY format (Mizuguchi et al., 1998). (E) Sequence alignment of DNA ligases. The figure shows a sequence alignment of the N-terminal of DBD and a DNA-binding region of NTD. The sequences with names of organisms are LigIV, and archaeal DNA ligases are labeled with PDB code in brackets. The residue numbers of the proteins are shown in brackets. Pink dots represent the VPF motif. K345 of human LigIV and equivalent residues of other ligases are indicated by a blue dot. (F) Comparison of the structures of $LigIV^{1-609}$ and DBD. The structures of the $LigIV^{1-609}$/$Artemis^{485-495}$ complex (the same color scheme as (A)), $LigIV^{1-609}$ (the same color scheme with the complex but that of DBD is magenta) and DBD (lime; PDB code: 4HTO) are superimposed based on residue 8-52 using Coot.

Supplemental Experiment Procedures

Protein expression and purification

Rossetta2(DE3)pLysS cells (Novagen) carrying the plasmid encoding LigIV$^{1-609}$ were grown at 37 ºC in 12 L of Terrific broth till OD_{600} reached 1.5 and incubated at 16 ºC overnight after being induced with 1 mM IPTG. The collected cells were frozen in liquid nitrogen and stored at -80 ºC. The frozen cells were re-suspended in the lysis buffer (50 mM Tris-HCl pH 8.0 at 4 ºC, 300 mM NaCl, 10 %(v/v) glycerol, 2 mM imidazole, 2 mM β-mercaptoethonal) supplied with cOmplete, EDTA-free protease inhibitor cocktail (Roche) and lysed by sonication.

The lysate was clarified by centrifuging the lysate at 27,000 g for 45 min before loading onto 20 ml of HIS-Select Nickel Affinity Gel (Sigma-Aldrich). The resin was washed with the lysis buffer before the bound proteins were eluted with the lysis buffer together with 300 mM imidazole. The eluate was dialysed against 5 L of the dialysis buffer (30 mM Tris-HCl pH 8.0 at 4 ºC, 300 mM NaCl, 5 %(v/v) glycerol, 2 mM β-mercaptoethonal) at 4 ºC overnight and then transferred to another 5 L of fresh dialysis buffer at 18 ºC after the sumo protease was added. The dialyzed solution was loaded onto 10 ml of HisTrap column (GE Healthcare) equilibrated with the dialysis buffer. The bound proteins were eluted with a linear gradient of 30 mM Tris-HCl pH 8.0 at 4 ºC, 300 mM NaCl, 5 %(v/v) glycerol, 2 mM β-mercaptoethonal, and 51 mM imidazole. The fractions containing LigIV$^{1-609}$ were collected and diluted two fold with 30 mM Tris-HCl pH 8.0 at 4 ºC, 5 %(v/v) glycerol, 5 mM DTT. The protein solution was then loaded onto 10 ml of Heparin Sepharose 6 Fast Flow equilibrated with a heparin buffer (30 mM Tris-HCl pH 8.0 at 4 ºC, 100 mM NaCl, 5 %(v/v) glycerol, 5 mM DTT), which was packed into a column. The bound proteins were eluted with a linear gradient of the heparin buffer and 1 M NaCl. The fractions containing LigIV$^{1-609}$ were diluted tenfold with a phenyl buffer (30 mM Tris-HCl pH 8.0 at 4 ºC, 1 M $(NH_4)_2SO_4$, 5 %(v/v) glycerol, 5 mM DTT) and loaded onto 5 ml HiTrap

phenyl HP (GE Healthcare) equilibrated with the phenyl buffer but 600 mM $(NH_4)_2SO_4$. The bound proteins were eluted with a linear gradient of 30 mM Tris-HCl pH 8.0 at 4 ºC, 100 mM NaCl, 5 %(v/v) glycerol, 5 mM DTT. The collected fractions were concentrated and loaded onto HiLoad 16/60 Superdex 200 prep grade (GE Healthcare) equilibrated with 20 mM Tris-HCl pH 8.0 at 4 ºC, 300 mM NaCl, 5 mM DTT. The peak fractions were collected and diluted threefold with 20 mM Tris-HCl pH 8.0 at 4 ºC, 5 mM DTT. Concentrated $LigIV^{1-609}$ was snap frozen in liquid nitrogen before stored at -80 ºC. The purified protein was confirmed as $LigIV^{1-609}$ by mass spectrometry conducted by Dr. Len Packman at the PNAC facility, Department of Biochemistry, University of Cambridge. $LigIV^{1-620}$ was purified using the same protocol.

The product protocol of JSB Floppy-Choppy (Jena Bioscience) was used for the limited proteolysis of $LigIV/XRCC4^{\Delta CTD;CtoA}$. The purification of $LigIV^{1-609}$ after proteolysis of $LigIV/XRCC4^{\Delta CTD;CtoA}$ was carried out as follows. 1.4 mg of the complex was incubated with an equal volume of 0.1 mg/ml subtilisin from JBS Floppy-Choppy at room temperature for 20 min. The volume of the solution was adjusted to 10 ml by 20 mM Tris-HCl pH 8.0 at 4 ºC, 200 mM NaCl, 5 %(v/v) glycerol, 5 mM DTT and loaded onto a 1 ml HiTrap heparin column HP (GE Healthcare). The bound proteins were eluted with a linear gradient of the same buffer but 650 mM NaCl for 25 ml. The fractions containing $LigIV^{1-609}$ were concentrated and loaded onto a Superdex 200 10/300 column (Pharmacia). Bands on SDS-PAGE gels were analyzed using mass spectroscopy by Dr. Len Packman and N-terminal sequencing by Mr. Mike Waldon at the PNAC facility.

Crystallization

The protein solution (120 μM $LigIV^{1-609}$, 144 μM $Artemis^{485-495}$, 144 μM 22 bp ligatable dsDNA, 1 mM ATP, 1 mM EDTA in 20 mM Tris-HCl pH 8.0 at 4 ºC, 100 mM NaCl, 5

mM DTT) or (120 µM LigIV$^{1-609}$, 10 mM $MgCl_2$, 1 mM ATP, in 20 mM Tris-HCl pH 8.0 at 4 ºC, 100 mM NaCl, 5 mM DTT) was incubated at 18 ºC for 60 min before crystallization using the hanging-drop vapor diffusion method. 1.2 µl of the solution was mixed with 0.8 µl of 100 mM MES pH 5.6 (for the complex) or 5.7 (for without Artemis$^{485-495}$), 2.0 M $(NH_4)_2SO_4$, 10 mM YCl_3 and 0.4 µl of a seeding stock. The drop was streaked on to a glass-covered slip instead of making a round drop, e.g. as in writing a letter “-“ or “Z”, before the slip was sealed over 700 µl of the crystallization solution. We eventually found that this method could reproduce crystallization of the proteins. The seeding stock was made by crushing single and multiple crystals of LigIV$^{1-609}$ in 10 µl of the reservoir solution using seed bead (Hampton Research).

DNA ligation assays

450 bp dsDNA was prepared by amplifying the *Saccharomyces cerevisiae* Lif1 gene using a forward primer (5'-GAGACACCATGGGCTCCCAGCTGACGGAG-3') and a reverse primer (5'-ATACGCGAATTCTCATTATCCCTTTTTGACTGGATG-3'). The PCR product was separated by electrophoresis in TBE gel and extracted. The substrate was digested by NcoI (NEB) and EcoRI (NEB). 1 pmol of proteins were incubated with 100 ng of the DNA ligation substrate in the ligation buffer (25 mM Tris-HCl pH 8.0 at room temperature, 50 mM KCl, 2.5 mM magnesium chloride, 1 mM DTT, 10 %(w/v) PEG 10,000, 0.5 mM ATP, 10 mg/ml BSA) at 37 °C for 5 and 30 min. 1/10 volume of the stop solution (0.1 %(w/v) SDS, 10 mM EDTA, 5 %(v/v) glycerol, 0.01 %(w/v) bromophenol blue) and 1/100 volume of 20 mg/ml Proteinase K (Melford) were added to the incubated samples, and were incubated at 37 °C for another 10 min (Modesti et al., 1999). Samples were separated by 0.8 % (w/v) TBE agarose electrophoresis in TBE, and stained and visualized with SYBR Gold (Invitrogen) and a UV imager.

Supplemental References

Edgar, R. C. (2004) MUSCLE: multiple sequence alignment with high accuracy and high throughput. Nucleic Acids Res. *32*, 1792-1797

Mizuguchi, K., Deane, C.M., Blundell, T.L., Johnson, M.S., and Overington, J.P. (1998). JOY: protein sequence-structure representation and analysis. Bioinformatics *14*, 617–623.

Modesti, M., Hesse, J. E. and Gellert, M. (1999). DNA binding of xrcc4 protein is associated with V(D)J recombination but not with stimulation of DNA ligase IV activity. EMBO J. *18*, 2008-2018.
